# Motor phenotype is not associated with vascular dysfunction in symptomatic Huntington’s disease transgenic R6/2 (160 CAG) mice

**DOI:** 10.1038/srep42797

**Published:** 2017-02-17

**Authors:** A. Di Pardo, A. Carrizzo, A. Damato, S. Castaldo, E. Amico, L. Capocci, M. Ambrosio, F. Pompeo, C. De Sanctis, C. C. Spinelli, A. A. Puca, P. Remondelli, V. Maglione, C. Vecchione

**Affiliations:** 1 IRCCS Neuromed, Pozzilli (Italy); 2 IRCCS Multimedica, Milano (Italy); 3 University of Salerno, Salerno (Italy)

## Abstract

Whereas Huntington’s disease (HD) is unequivocally a neurological disorder, a critical mass of emerging studies highlights the occurrence of peripheral pathology like cardiovascular defects in both animal models and humans. The overt impairment in cardiac function is normally expected to be associated with peripheral vascular dysfunction, however whether this assumption is reasonable or not in HD is still unknown. In this study we functionally characterized the vascular system in R6/2 mouse model (line 160 CAG), which recapitulates several features of human pathology including cardiac disease. Vascular reactivity in different arterial districts was determined by wire myography in symptomatic R6/2 mice and age-matched wild type (WT) littermates. Disease stage was assessed by using well-validated behavioural tests like rotarod and horizontal ladder task. Surprisingly, no signs of vascular dysfunction were detectable in symptomatic mice and no link with motor phenotype was found.

Huntington’s disease (HD) is one of the most common non-curable rare diseases characterized primarily by a progressive loss of cognitive and motor function leading to severe disability and death in affected patients^1^. Expansion of the trinucleotide repeat (CAG) within the huntingtin (HTT) gene is recognized as the major cause^2^ and its length is known to profoundly influence age at onset and disease phenotype^3^,^4^. Whereas HD is unequivocally a neurological disorder, there is a critical mass of emerging studies suggesting peripheral pathology as an important factor that might significantly contribute to the overall presentation and progression of the disease.

Interestingly, multiple epidemiological studies report evidence of heart pathology in HD and describe cardiac failure as one of the more common causes of death among disease patients^5^,^6^. Subtle abnormalities of autonomic control of the cardiovascular system in HD have already been reported at pre-symptomatic and early stage^7^,^8^ and described to gradually progress in range and magnitude as the disease advances^9^. Similar dysfunctional cardiac phenotype has been observed also in pre-clinical HD settings^10^,^11^,^12^, however still much is needed to fully understand whether there exists any direct association with peripheral vascular function. Despite the available studies examining this possible correlation in HD, there is currently insufficient evidence to make a definitive conclusion.

While alterations in the structure of vascular network have clearly been implicated in brain pathology in HD^13^, definitive evidence of vascular homeostasis is still lacking.

Aside from few studies reporting only partial evidence of deranged peripheral vascular function in some of the available HD models^14^,^15^, no comprehensive study that systematically investigated the functional vascular reactivity has been conducted so far.

To this regard, here we sought to provide a more complete profile of vascular function and contractile properties of either resistance or capacitance vessels in both central and peripheral districts in R6/2 mouse model (160 CAG), the better characterized and the most used model when studying cardiac function in HD^11^,^12^,^13^,^15^, which recapitulates several features of motor and behavioural phenotype of early human pathology^16^,^17^.

In this study, our thorough investigation of symptomatic 12 week old R6/2 mice has led to the first full characterization of vascular function in this model, in which we could not detect any vascular dysfunction or molecular defects in possible related signalling pathways like the one involving the synthesis of Nitric Oxide (NO), whose dysfunction has been previously hypothesized to pre-date the disease manifestation in HD models^15^.

## Methods

### Animal model

Transgenic HD R6/2 line, expressing exon 1 of the human huntingtin gene carrying approximately 160 +/− 5 GAC repeat expansions, was originally purchased from Jackson Laboratories (Bar Harbor, ME, USA) and the colony was maintained by breeding heterozygous R6/2 males with wild-type (WT) females from their background strain (B6CBA-Tg(HDexon)62oGpb/J) in the animal facility at IRCCS Neuromed. Genotyping was confirmed by PCR and performed at 3 weeks of age to determine study groups. Mixed gender F1 mouse generation was used in this study. Animals were housed in polycarbonate cages (15 × 23 × 17 cm) provided with a mouse house and aspen bedding and maintained under temperature (22–24 °C) and humidity-controlled (55%) conditions. Food and water were provided *ad libitum*.

All efforts were made to minimize the number of animals used and their suffering. All the experiments reported in this study were performed on the same animal groups. All animal procedures were conformed to the guidelines for the Care and Use of Laboratory Animals published from Directive 2010/63/EU of the European Parliament and approved by the IRCCS Neuromed Animal Care Review Board and by “Istituto Superiore di Sanità” (permit number: 1163/2015- PR).

### Assessment of motor function and disease progression in R6/2 mice

Fine-motor skills and coordination were performed using well-validated motor tests according to the standard recommendations. All tests took place during the light phase of the light–dark cycle. Six mice per experimental group were used in each test. All mice received training for 2 consecutive days on each instrument and task before performing motor behavior measurements. Before training and testing, mice underwent a period of habituation to the testing room and equipment. Motor coordination and balance were tested on the rotarod apparatus as previously described^18^. Briefly, mice were tested at fixed speed (20 rpm) on the rotarod (Ugo Basile) for 1 min. Each mouse was tested in three consecutive trials of 1 min each, with 1 min rest between trials. The time spent on the rotarod in each of the three trials was averaged to give the overall time for each mouse.

Skilled walking, limb placement and limb coordination were all assessed by the ladder rung walking task as previously described^18^. All tests were carried out once per week until the 11th week of age. Concomitant with the analysis of motor performance, animal body weight was also measured. All mouse cages were daily examined in order to determine disease progression and the overall wellbeing of mice.

### Blood pressure measurements

Blood pressure was measured using the BP-2000 instrument (Visitech systems). The tail cuff method was carried out as described previously^19^. After four days training period, basal systolic and diastolic blood pressure was daily measured for one week in conscious and unrestrained R6/2 mice at different time points during the symptomatic stage of the disease (7, 10 and 12 week old mice) and in age-matched WT littermates.

### *Ex vivo* vascular reactivity in resistance vessels

Vascular reactivity studies were carried out in second-order branches of the mesenteric arterial tree or in femoral arteries from symptomatic HD mice (12 week old) and age-matched WT littermates.

Briefly, vessels were excissed from mice and adventitial fat was carefully removed under a dissection microscope (Nikon, SMZ645). Arteries were then mounted on pressure myograph (DMT Danish Myosystem) filled with Krebs solution (pH 7.4) maintained at 37 °C as previously described^19^. After an equilibration period of 60 minutes, vasoconstrictive response was assessed in presence of 80 mM KCl or in presence of increasing doses of phenylephrine (1 × 10^−9^ to 10^−5^ M) until a plateau was reached. Vessels were then washed at least three times in order to stabilize the vascular tissue. Endothelium-dependent and -independent relaxations were assessed in phenylephrine pre-constricted vessels by measuring the vasorelaxant response to cumulative concentrations of acetylcholine (1 × 10^−9^ to 10^−5^ M) or nitroglycerine (1 × 10^−9^ to 10^−5^ M), respectively. Moreover, in order to assess the contribution of NO-signaling to vascular function in our symptomatic HD mice, mesenteric arteries from both WT and R6/2 mice were pre-treated with the direct NOS inhibitor N^G^-nitro-L-arginine methyl ester (L-NAME, 300 μM, 30 min) before performing the analysis of acetylcholine*-*induced vasorelaxation.

### *Ex vivo* vascular reactivity in capacitance vessels

To test the vascular response in the capacitance vessels from symptomatic HD mice (12 week old) and age-matched WT littermates, we studied aorta and carotid arteries. In detail, after the excision of vessels from mice, fat tissue was careful removed and vessels cross-sectioned into 2 mm long rings. Two stainless steel wires were inserted into the vascular lumen of aorta, placed in a chamber and connected to a force transducer (WPI). Carotid arteries were mounted in a wire myograph (model 410 A, Danish MyoTechnogy, Aarhus, Denmark) over 25-μm tungsten wires and placed in organ baths filled with aerated Krebs solution connected to a force transducer. After an equilibration period of 60 minutes, vasoconstrictive response was assessed with 80 mM KCl or with increasing doses of phenylephrine (1 × 10^−9^ to 10^−5^ M) until a plateau was reached. Vascular response of phenylephrine pre-constricted vessels, to cumulative concentrations of acetylcholine and nitroglycerin was examined to determine the endothelium-dependent and -independent relaxation, respectively.

### Immunoblotting

After isolation, arteries were solubilized in lysis buffer containing 20 mmol/L Tris-HCl, 150 mmol/L NaCl, 20 mmol/L NaF, 2 mmol/L sodium orthovanadate, 1% Nonidet, 100 μg/ml leupeptin, 100 μg/ml aprotinin and 1 mmol/L phenylmethylsulfonyl fluoride. Samples were left on ice for 30 minutes, centrifuged at 10000 *g* for 15 minutes and supernatants were used to perform Western immunoblot analysis. Total protein levels were determined using the Bradford method. 30 μg proteins were resolved on 7% SDS-PAGE, transferred to a nitrocellulose membrane and immunoblotted with anti-phospho-eNOS S1177 (Cell Signaling, rabbit polyclonal antibody 1:800) and with anti-total-eNOS (Cell Signaling, mouse mAb 1:1000). HRP-conjugated secondary antibodies were used at 1:3000 dilution (Bio-Rad Laboratories). Protein bands were detected by ECL Prime (Amersham Biosciences) and quantitated with Quantity One software (Bio-Rad Laboratories).

### Statistics

All data were expressed mean ± standard error of mean (SEM). Data were statistically analyzed by two-way ANOVA followed by Bonferroni post-hoc analysis, using a dedicated software (GraphPad Prism Software, version 5.0).

## Results

### Motor performance and disease progression in R6/2 mice

**Figure 1:**
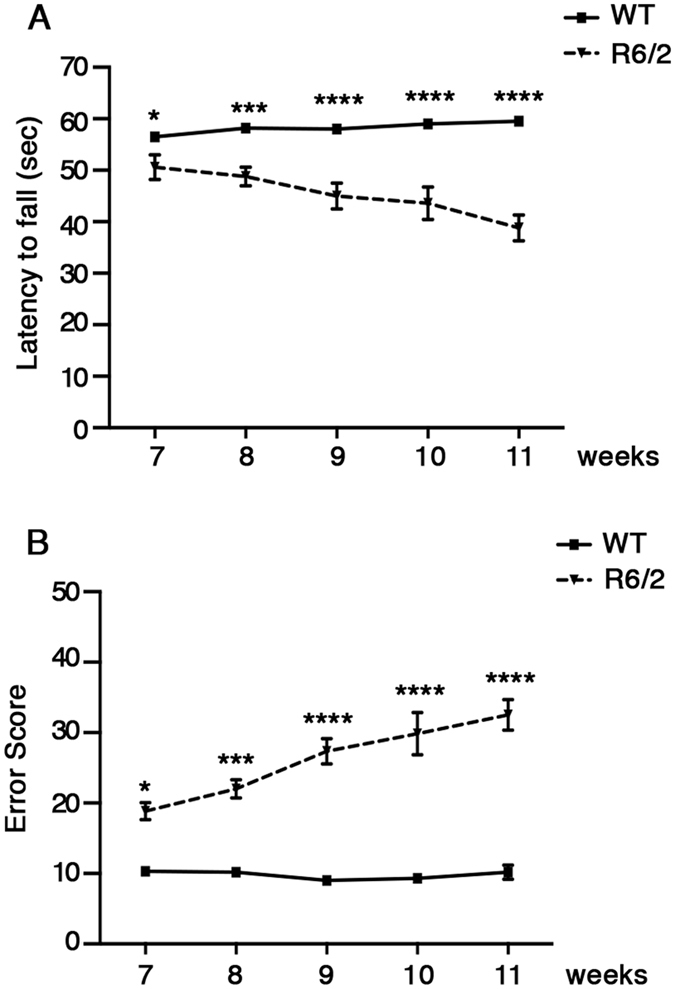
Analysis of motor coordination in HD mice. (**A**) Latency to fall on the Rotarod and (**B**) total error score on the Horizontal Ladder Task in symptomatic R6/2 mice and age-matched WT littermates. Each data point represents the average performance ± S.E.M. of 6 mice for each group. *P < 0.05; ***P < 0.001; ****P < 0.0001. Two-way ANOVA with Bonferroni post-test.

Rigorous evaluation of motor coordination to monitor disease progression and to precisely determine the advanced disease stage in our R6/2 line was performed using standardized procedures as previously described^18^. As expected, HD mice developed a measurable neurological phenotype by 7–8 weeks of age coherently with the original report^16^ and, displayed a marked and progressive deterioration in motor performance when compared to WT mice (Fig. 1A,B) as the disease progressed. The severity of these abnormalities worsened gradually until 11 weeks of age, when the advanced disease stage-dependent neurological deterioration classically occurring in this model^20^, irreversibly affected the overall wellbeing of our mice (Two-way ANOVA. Rotarod: interaction, F (4, 40) = 8.796; p < 0.0001. Horizontal Ladder Task: interaction, F (4, 40) = 8.210; p < 0.0001) (Fig. 1 and Supplementary Tables 1 and 2). As expected, body weight was gradually decreased in R6/2 mice as the disease progressed when compared with age-matched WT littermates (Supplementary Figure 1).

### Arterial blood pressure in R6/2 mice

**Figure 2:**
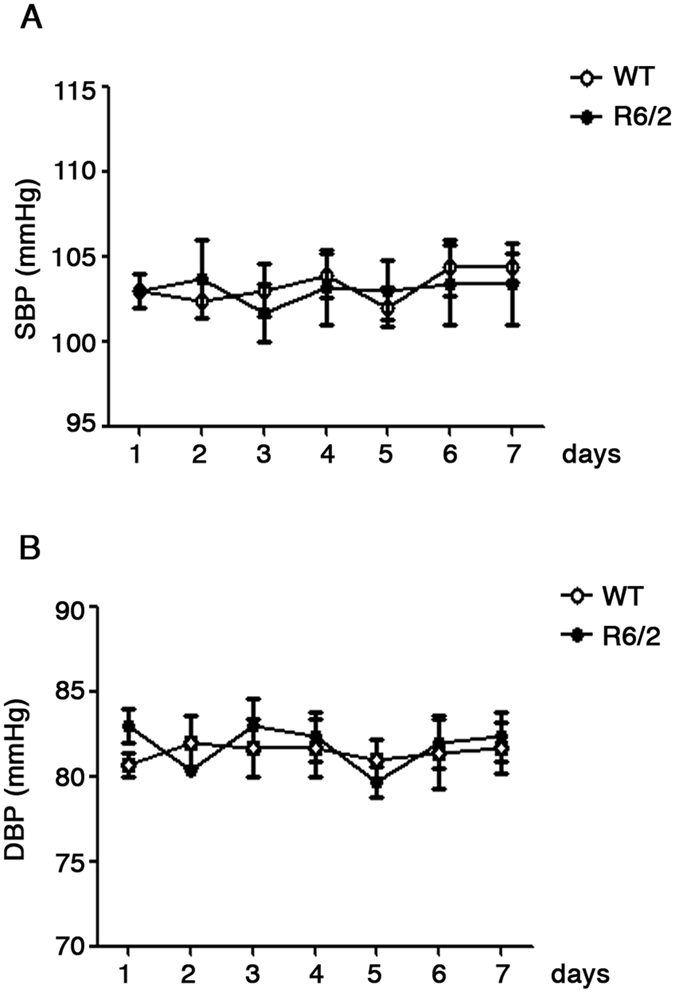
Measurement of blood pressure in HD mice. Systolic (SBP) (**A**) and Diastolic blood pressure (DBP) in 12 week old R6/2 mice and age-matched WT littermates. Data are expressed as mean per ± S.E.M. of 6 mice for each group. Two-way ANOVA with Bonferroni post-test.

With the aim of delineating a clearer picture of cardiovascular homeostasis in our HD model, measurement of arterial blood pressure was performed using tail-cuff technique in symptomatic R6/2 mice (7,10 and 12 weeks of age) and in age-matched WT controls. Average values of both systolic and diastolic arterial blood pressure, ranging from 85 to 105 mmHg, was not significantly different between the two groups across all the stages of the disease (Two-way ANOVA. SBP; F (6,35) = 0,4336; p = 0.8514; DBP; F(6,35) = 1,443; p = 0.2264) (Fig. 2A,B and Supplementary Figure 2A-B).

### Arteries vascular function

In order to fully characterize the functional features of the vasculature in HD, vascular reactivity of both resistance and capacitance arteries from either R6/2 or WT mice at 12 weeks of age was systematically assessed.

**Figure 3:**
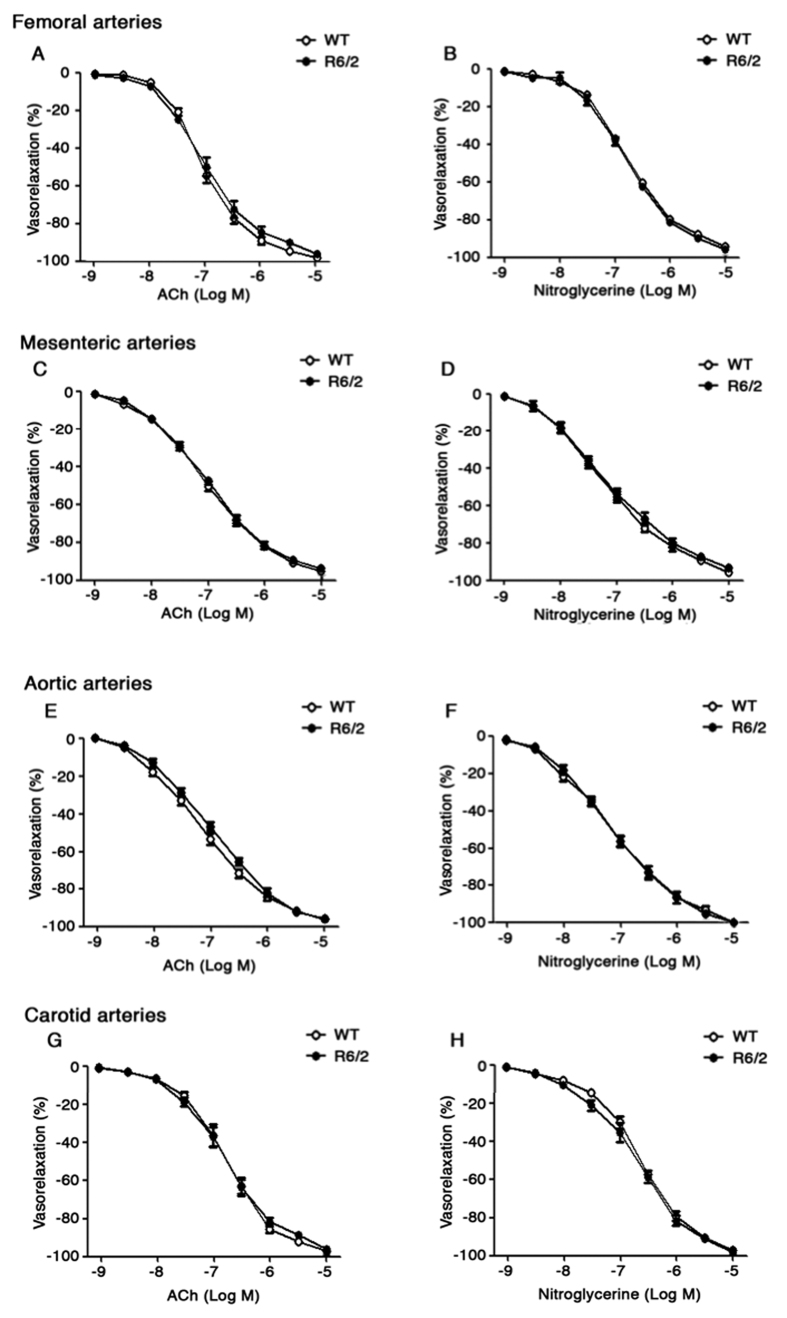
Analysis of vasorelaxation in HD mice. Dose–response curves of phenylephrine-pre-contracted femoral (**A,B**), mesenteric (**C,D**), aortic (**E,F**) and carotid (**G,H**) arteries in response to acetylcholine (ACh) and nitroglycerine in symptomatic R6/2 mice and age-matched WT littermates. Data are expressed as mean per ± S.E.M. of 9 mice for each group. Two-way ANOVA with Bonferroni post-test.

**Figure 4:**
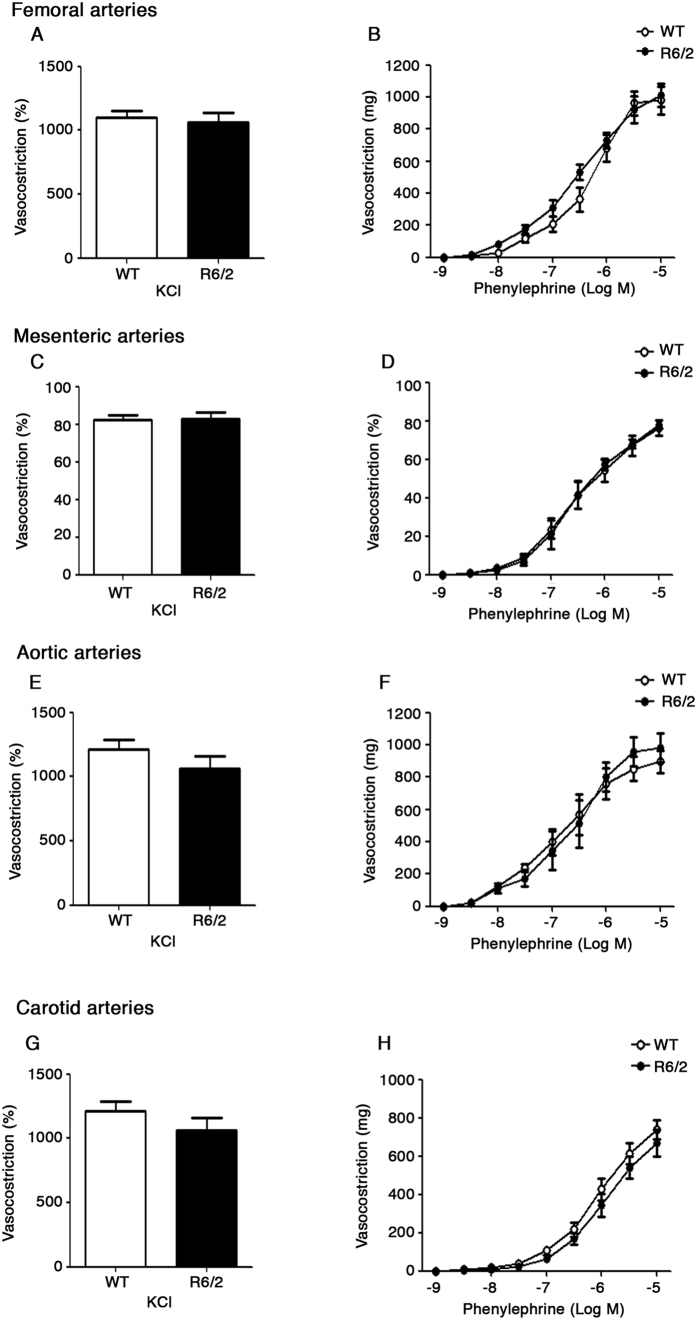
Analysis of vasocostriction in HD mice. Vascular response of femoral (**A**,**B**), mesenteric (**C**,**D**), aortic (**E**,**F**) and carotid (**G**,**H**) arteries in response to KCL and Phenylephrine in symptomatic R6/2 mice and age-matched WT littermates. Data are expressed as mean per ± S.E.M. of 6 mice for each group. Two-way ANOVA with Bonferroni post-test.

Analysis of vasodilator function in phenylephrine pre-contracted femoral (Fig. 3A,B) and mesenteric (Fig. 3C,D) arteries as well as in aortic (Fig. 3E,F) and carotid (Fig. 3G,H) arteries in response to the endothelium-dependent agonist, acetylcholine, and to the endothelium-independent agonist, nitroglycerine, showed no difference between the two genotypes and no signs of vascular dysfunction was detectable in any of the districts analyzed (Two-way ANOVA. Femoral arteries; ACh; F (8,128) = 1,698; p = 0.1048; Nitro; F (8,128) = 0,5216; p = 0.8385; Mesenteric arteries; ACh; F(8,128) = 0,3317; p = 0.9524; Nitro; F(8,128) = 0,3037; p = 0.9634; Aorta; ACh; F (8,128) = 1,379; p = 0.2116; Nitro; F (8,128) = 0,3382; p = 9496. Carotid arteries; ACh; F (8,128) = 0,4320, p = 0.9000; Nitro; F (8,128) = 0,8280; p = 0.5794) (Fig. 3). Consistently with the findings of preserved vasodilator function in advanced disease stage, analysis of vasocostrictor function in response to either potassium or to the α1-adrenergic agonist, phenylephrine, consolidated the evidence of “normal” vascular reactivity in all vessels analyzed (Two-way ANOVA. Femoral arteries; KCl; F (1,5) =  0,5177; p = 0.5040; Phenylephrine; F (8,80) = 0,3364; p = 0.9493. Mesenteric arteries; KCl; F (1,5) = 0,6849; p = 0.4456; Phenylephrine; F (8,80) =  0,3482; p = 0,9440. Aorta; KCl; F (1,5) = 1,777; p = 0,2400; Phenylephrine; F(8,80) =  1,16; p = 0,3335. Carotid arteries; KCl; F (1,5) = 0,2959; p = 6098; Phenylephrine; F (8,80) = 1,589; p = 0.1413) (Fig. 4).

### eNOS signaling pathway in advanced stages of HD pathology in R6/2 mice

**Figure 5:**
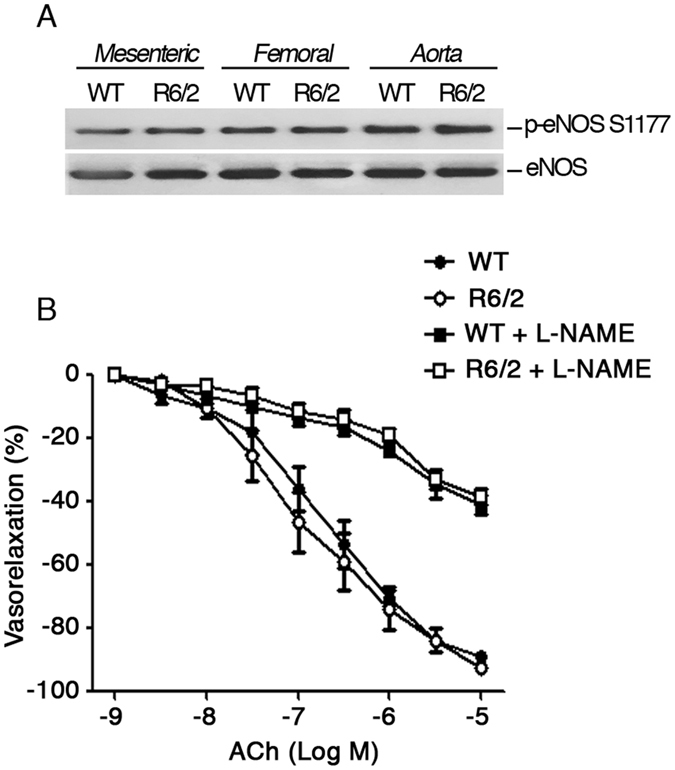
Molecular contribution of NO-signaling to vascular function in HD mice. (**A**) Representative immunoblotting of eNOS phosphorylation at serine residue 1177 and eNOS protein levels in Mesenteric, Femoral and Aortal arteries in 12 week old R6/2 mice and WT Littermate. **(B)** Dose–response curves of phenylephrine-pre-contracted mesenteric arteries in presence or absence of L-NAME in 12 week-old R6/2 and age-matched WT littermates. N = 6. Two-way ANOVA with Bonferroni post-test. Data are expressed as mean per ± S.E.M.

Nitric oxide is the main determinant of the endothelium-mediated vasorelaxant effects and normally synthesized by the endothelial Nitric Oxide Synthase (eNOS)^21^,^22^,^23^, whose phosphorilation at Ser 1177 residue directly enhances enzyme activity^23^. Despite the normal vascular function in arteries from symptomatic R6/2 mice, here we investigated whether eNOS pathway was indeed perturbed. Coherently with the evidence of unchanged vasorelaxant responses in advanced stage of HD (Fig. 3), no difference in either eNOS phosphorylation state or protein expression was observed between WT and R6/2 mice (Fig. 5). At functional level, the selective inhibition of NOS by L-NAME blunted acetylcholine-induced vasorelaxation to the same extent in both WT and R6/2 mice and further confirmed the lack of significant difference in the NO-dependent vasorelaxation in both animal groups (Two-way (Two-way ANOVA. F (24,160) =36,88; p < 0,0001) (Fig. 5B).

## Discussion

It is now well established that HD pathology is not only confined to the central nervous system (CNS), but rather comprises a large group of peripheral defects including cardiac phenotype^24^. In agreement with the evidence that neurological diseases are positively linked to cardiovascular pathology and that CNS abnormalities have a great impact on the pathogenesis of cardiac dysfunction^25^, it has been postulated that also HD-related cardiac alterations are likely driven by CNS dysfunctions. However, whether there exists any physiopathological interdependence between the progressive disease phenotype and the vascular function in HD has never been assessed before.

While significant cardiac phenotype has been extensively described to typify HD pathology in both human patients^26^ and animal models^27^,^28^, no definitive characterization of vascular function has been exhaustively performed so far. The only comprehensive study that has partially addressed this issue, has shown a defective contractility of some of the systemic arteries only at very advanced age and disease stages of transgenic R6/1 mice^14^, a HD mouse model with late age at onset, slow disease progression and high life expectancy^16^. Similar investigation has been recently performed by Kane *et al*., in a sub-line of R6/2 mouse model which displays a prolonged disease progression and longer lifespan^15^ than prototypical parent R6/2 mice^16^,^17^,^29^, the most extensively studied and utilized mouse model of HD. Although a derangement in peripheral vascular reactivity has been described, the study of Kane *et al*., limited the analysis only to the femoral arteries and partially described the vascular phenotype these mice can display^15^.

Here, with the aim of providing a more detailed functional characterization in HD, an accurate and systematic investigation into vascular reactivity in both central and peripheral arteries was performed in symptomatic parental R6/2 model^16^,^17^,^29^, which differs from that used by Kane *et al*., in CAG repeat length (160 ± 5 CAG vs 242 ± 1 CAG) and life expectancy (12–13 weeks of age vs. 24 weeks of age)^11^,^16^,^30^.

Analysis of vascular function in our R6/2 line failed to reveal any difference between HD and control mice in all districts analyzed. Moreover, the unaffected vascular reactivity was also accompanied by regular blood pressure profile and unchanged endothelial-mediated NO vasorelaxation. The lack of any alteration in the endothelial function in our symptomatic R6/2 mice was corroborated by unperturbed eNOS signaling in vessels from the same mice. Curiously, our findings did not found full support on the previous study^15^. The reason for this discrepancy is not clear, however it could be likely attributable, at least for the vasoconstrictor function, to the different genotypes (CAG length) and the dependent and inverted U-shaped profile of disease progression and life expectancy^31^. On the other hand, although apparently different, endothelial function showed a comparable vasodilator profile in HD mice in both mouse lines at 12 weeks of age. The endothelial dysfunction described by Kane and collaborators in control mice at similar age, and its improvement with aging was rather unexpected.

In this study, the overall absence of vascular dysfunction in our symptomatic R6/2 mice does not exclude that such a specific phenotype may develop later in the disease, that in the case of this mouse line is unlikely to happen because of the characteristic short lifespan and high rate of early death that can occur before peripheral perturbations appear. This hypothesis is also supported by the evidence of impaired vascular function only in aged diseased animal model^14^. From our perspective, the perfect overlapping profile of vascular reactivity and blood pressure as well as the lack of any change at molecular levels between WT and HD mice definitively bypasses the relative small sample size that could normally represent a limitation.

To our knowledge, this study presents the first full characterization of vascular function in HD transgenic R6/2 mice (160 ± 5 CAG).

Collectively, the combination of functional and biochemical experiments highlights a normal vascular phenotype in these mice and indicates that motor abnormalities do not depend on vascular dysfunction in our mouse model. Therefore, we can conclude that our findings delineate limitations on the usefulness of this mouse line when studying certain aspect associated with aging during the progression of the disease.

## Additional Information

**How to cite this article:** Di Pardo, A. *et al*. Motor phenotype is not associated with vascular dysfunction in symptomatic Huntington’s disease transgenic R6/2 (160 CAG) mice. *Sci. Rep.* **7**, 42797; doi: 10.1038/srep42797 (2017).

**Publisher's note:** Springer Nature remains neutral with regard to jurisdictional claims in published maps and institutional affiliations.

## Supplementary Material

Supplementary Information

## References

[b1] Bates G., Harper P. S. & Jones L. Huntington’s Disease. Oxford University Press 3rd edn, 3–27 (2002).

[b2] Kremer B. . A worldwide study of the Huntington’s disease mutation. The sensitivity and specificity of measuring CAG repeats. N. Engl. J. Med. 330, 1401–1406 (1994). 8159192 10.1056/NEJM199405193302001

[b3] Falush D., Almqvist E. W., Brinkmann R. R., Iwasa Y. & Hayden M. R. Measurement of mutational flow implies both a high new-mutation rate for Huntington disease and substantial underascertainment of late-onset cases. Am. J. Hum. Genet. 68, 373–385 (2000). 10.1086/318193 PMC1235271 11225602

[b4] Walker F. O. Huntington’s disease. Lancet 369, 218–228 (2007). 17240289 10.1016/S0140-6736(07)60111-1

[b5] Chiu E. & Alexander L. Causes of death in Huntington’s disease. Med J Aust. 1, 153 (1982). 10.5694/j.1326-5377.1982.tb132224.x 6210834

[b6] Lanska D. J., Lavine L., Lanska M. J. & Schoenberg B. S. Huntington’s disease mortality in the United States. Neurology 38, 769–772 (1988). 2966305 10.1212/wnl.38.5.769

[b7] Aziz N. A., Anguelova G. V., Marinus J., van Dijk J. G. & Roos R. A. Autonomic symptoms in patients and pre-manifest mutation carriers of Huntington’s disease. Eur J Neurol. 8, 1068–74 (2010). 10.1111/j.1468-1331.2010.02973.x 20192977

[b8] Kobal J. . Autonomic dysfunction in presymptomatic and early symptomatic Huntington’s disease. Acta Neurol Scand. 121, 392–399 (2010). 20047567 10.1111/j.1600-0404.2009.01251.x

[b9] Andrich J. . Autonomic nervous system function in Huntington’s disease. J Neurol Neurosurg Psychiatry 72, 726–31 (2002). 12023413 10.1136/jnnp.72.6.726 PMC1737927

[b10] Schroeder A. M. . Cardiac Dysfunction in the BACHD Mouse Model of Huntington’s Disease. PLoS One 11, e0147269 (2016). 26807590 10.1371/journal.pone.0147269 PMC4725962

[b11] Wood N. I. . Direct evidence of progressive cardiac dysfunction in a transgenic mouse model of Huntington’s disease. J Huntingtons Dis. 1, 57–64 (2012). 24339845 10.3233/JHD-2012-120004 PMC3856869

[b12] Mihm M. J. . Cardiac dysfunction in the R6/2 mouse model of Huntington’s disease. Neurobiol Dis 25, 297–308 (2007). 17126554 10.1016/j.nbd.2006.09.016 PMC1850107

[b13] Drouin-Ouellet J. . Cerebrovascular and blood-brain barrier impairments in Huntington’s disease: Potential implications for its pathophysiology. Ann Neurol. 78, 160–77 (2015). 25866151 10.1002/ana.24406

[b14] Rahman A. . Late onset vascular dysfunction in the R6/1 model of Huntington’s disease. Eur J Pharmacol. 698, 345–53 (2013). 23117088 10.1016/j.ejphar.2012.10.026

[b15] Kane A. D., Niu Y., Herrera E. A., Morton A. J. & Giussani D. A. Impaired Nitric Oxide Mediated Vasodilation In The Peripheral Circulation In The R6/2 Mouse Model Of Huntington’s Disease. Sci Rep. 6, 25979 (2016). 27181166 10.1038/srep25979 PMC4867587

[b16] Mangiarini L. . Exon 1 of the HD gene with an expanded CAG repeat is sufficient to cause a progressive neurological phenotype in transgenic mice. Cell. 87, 493–506 (1996). 8898202 10.1016/s0092-8674(00)81369-0

[b17] Carter R. J. . Characterization of progressive motor deficits in mice transgenic for the human Huntington’s disease mutation. J Neurosci. 19, 3248–57 (1999). 10191337 10.1523/JNEUROSCI.19-08-03248.1999 PMC6782264

[b18] Di Pardo A. . Ganglioside GM1 induces phosphorylation of mutant huntingtin and restores normal motor behavior in Huntington disease mice. Proc Natl Acad Sci USA 109, 3528–33 (2012). 22331905 10.1073/pnas.1114502109 PMC3295265

[b19] Carrizzo A. . Morus alba extract modulates blood pressure homeostasis through eNOS signaling. Mol Nutr Food Res. 60, 2304–2311 (2016). 27234065 10.1002/mnfr.201600233

[b20] Li J. Y., Popovic N. & Brundin P. The use of the R6 transgenic mouse models of Huntington’s disease in attempts to develop novel therapeutic strategies. NeuroRx. 2, 447–64 (2005). 16389308 10.1602/neurorx.2.3.447 PMC1144488

[b21] Aggio A. . Endothelium/nitric oxide mechanism mediates vasorelaxation and counteracts vasoconstriction induced by low concentration of flavanols. Eur J Nutr. 52, 263–72 (2013). 22322926 10.1007/s00394-012-0320-x

[b22] Yamashita T. . Mechanisms of Reduced Nitric Oxide/cGMP–Mediated Vasorelaxation in Transgenic Mice Overexpressing Endothelial Nitric Oxide Synthase. Hypertension 36, 97–102 (2000). 10904019 10.1161/01.hyp.36.1.97

[b23] Carrizzo A. . Nitric Oxide Dysregulation in Platelets from Patients with Advanced Huntington Disease. PLoS One 9, e89745 (2014). 24587005 10.1371/journal.pone.0089745 PMC3934931

[b24] Mielcarek M. Huntington’s disease is a multi-system disorder. Rare Dis. 3, e1058464 (2015). 26459693 10.1080/21675511.2015.1058464 PMC4588536

[b25] Shen M. J. & Zipes D. P. Role of the autonomic nervous system in modulating cardiac arrhythmias. Circ Res. 114, 1004–1021 (2014). 24625726 10.1161/CIRCRESAHA.113.302549

[b26] Abildtrup M. & Shattock M. Cardiac Dysautonomia in Huntington’s Disease. J Huntingtons Dis. 2, 251–61 (2013). 25062674 10.3233/JHD-130054

[b27] Kiriazis H. . Neurocardiac dysregulation and neurogenic arrhythmias in a transgenic mouse model of Huntington’s disease. J Physiol. 590, 5845–60 (2012). 22890713 10.1113/jphysiol.2012.238113 PMC3528995

[b28] Mielcarek M. . Dysfunction of the CNS-heart axis in mouse models of Huntington’s disease. PLoS Genet. 10, e1004550 (2014). 25101683 10.1371/journal.pgen.1004550 PMC4125112

[b29] Cummings D. M. . A critical window of CAG repeat-length correlates with phenotype severity in the R6/2 mouse model of Huntington’s disease. J Neurophysiol. 107, 677–91 (2012). 22072510 10.1152/jn.00762.2011 PMC3349621

[b30] Di Pardo A. . FTY720 (fingolimod) is a neuroprotective and disease-modifying agent in cellular and mouse models of Huntington disease. Hum Mol Genet. 23, 2251–65 (2014). 24301680 10.1093/hmg/ddt615

[b31] Tang B. . Gene expression profiling of R6/2 transgenic mice with different CAG repeat lengths reveals genes associated with disease onset and progression in Huntington’s disease. Neurobiol Dis. 42, 459–67 (2011). 21334439 10.1016/j.nbd.2011.02.008 PMC3079804

