# Supplementary material for: Motor phenotype is not associated with vascular dysfunction in symptomatic Huntington’s disease transgenic R6/2 (160 CAG) mice
Source: Sci Rep. 2017 Feb 17;7:42797. doi: 10.1038/srep42797 (PMC5314343; doi:10.1038/srep42797)
Supplement: Supplementary Information [file srep42797-s1.pdf]

Supplementary Information

Motor phenotype is not associated with vascular dysfunction in symptomatic Huntington's disease transgenic R6/2 (160 CAG) mice.

Di Pardo A[1], Carrizzo A[1], Damato A[1], Castaldo S[1], Amico E[1], Capocci L[1], Ambrosio M[1], Pompeo F[1], De Sanctis C[1], Puca AA[2,3], Remondelli P[3], Maglione V[1*] and Vecchione C[1,3*].

[1] IRCCS Neuromed, Pozzilli (Italy) [2] IRCCS Multimedica, Milano (Italy) [3] University of Salerno, Salerno (Italy)

*Correspondence to:
Carmine Vecchione, IRCCS Neuromed,
Vascular Physiopathology Unit,
Loc. Camerelle, 86077, Pozzilli (IS), Italy.
University of Salerno,
Department of Medicine and Surgery,
via Allende, 84084, Baronissi (SA), Italy.
E-mail cvecchione@unisa.it
and
Vittorio Maglione, IRCCS Neuromed,
Centre for Neurogenetics and Rare Disease,
Loc. Camerelle, 86077, Pozzilli (IS), Italy.
E-mail: vittorio.maglione@neuromed.it

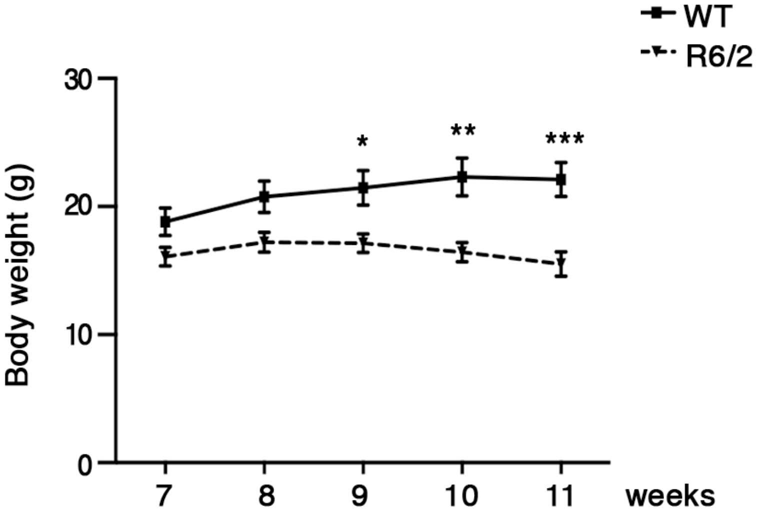

Supplementary Figure 1. Body weight. Body weight measurement in symptomatic R6/2 mice and WT littermates. Each data point represents the average performance ± S.E.M. of 6 mice for each group. *, P<0.05; **, *P* < 0.001; ***, *P* < 0.0001. Two-way ANOVA with Bonferroni post-test. Interaction, $F (4, 40) = 34.27$; $p<0.0001$. Complete tabular statistics for ANOVA is reported in the Supplementary Table 2.

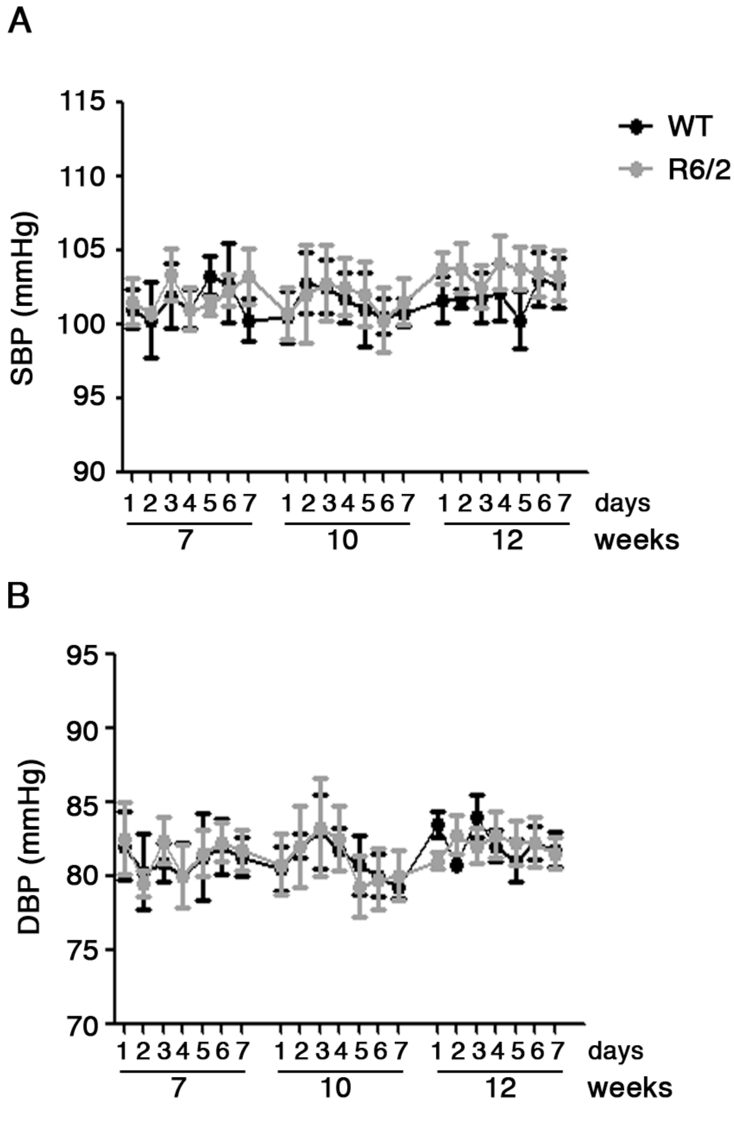

Supplementary Figure 2. Measurement of blood pressure in HD mice. Systolic (SBP) (A) and Diastolic blood pressure (DBP) in 7, 10 and 12 weeks old R6/2 mice and age-matched WT littermates. N=6 for each groups. Two-way ANOVA, Bonferroni post-test. SBP; F= 0,6037. DBP; F= 0,5026

Supplementary Table 1. Tabular results for Two Way ANOVA reported in Figure 1

2way ANOVA<br>Tabular results					
Table Analyzed	Rotarod				
Two-way RM ANOVA	Matching: Stacked				
Alpha	0.05				
Source of Variation	% of total variation	P value	P value summary	Significant?	
Interaction	4.407	< 0.0001	****	Yes	
weeks	1.511	0.0290	*	Yes	
genotype	68.00	0.0002	***	Yes	
Subjects (matching)	21.07	< 0.0001	****	Yes	
ANOVA table	SS	DF	MS	F (DFn, DFd)	P value
Interaction	182.7	4	45.69	F (4, 40) = 8.796	P < 0.0001
weeks	62.64	4	15.66	F (4, 40) = 3.015	P = 0.0290
genotype	2820	1	2820	F (1, 10) = 32.28	P = 0.0002
Subjects (matching)	873.7	10	87.37	F (10, 40) = 16.82	P < 0.0001
Residual	207.8	40	5.194		
Number of missing values	0				

2way ANOVA<br>Tabular results					
Table Analyzed	Horizontal				
Two-way RM ANOVA	Matching: Stacked				
Alpha	0.05				
Source of Variation	% of total variation	P value	P value summary	Significant?	
Interaction	8.710	< 0.0001	****	Yes	
weeks	6.049	0.0010	***	Yes	
genotype	69.17	< 0.0001	****	Yes	
Subjects (matching)	5.466	0.0519	ns	No	
ANOVA table	SS	DF	MS	F (DFn, DFd)	P value
Interaction	428.8	4	107.2	F (4, 40) = 8.210	P < 0.0001
weeks	297.8	4	74.44	F (4, 40) = 5.701	P = 0.0010
genotype	3405	1	3405	F (1, 10) = 126.6	P < 0.0001
Subjects (matching)	269.1	10	26.91	F (10, 40) = 2.061	P = 0.0519
Residual	522.3	40	13.06		
Number of missing values	0				

Supplementary Table 2. Tabular results for Two Way ANOVA reported in Supplementary Figure 1

2way ANOVA<br>Tabular results					
Table Analyzed	Body Weight				
Two-way RM ANOVA	Matching: Stacked				
Alpha	0.05				
Source of Variation	% of total variation	P value	P value summary	Significant?	
Interaction	4.215	< 0.0001	****	Yes	
weeks	3.983	< 0.0001	****	Yes	
genotype	43.61	0.0123	*	Yes	
Subjects (matching)	46.97	< 0.0001	****	Yes	
ANOVA table	SS	DF	MS	F (DFn, DFd)	P value
Interaction	30.83	4	7.707	F (4, 40) = 34.27	P < 0.0001
weeks	29.14	4	7.284	F (4, 40) = 32.39	P < 0.0001
genotype	319.0	1	319.0	F (1, 10) = 9.285	P = 0.0123
Subjects (matching)	343.5	10	34.35	F (10, 40) = 152.7	P < 0.0001
Residual	8.997	40	0.2249		
Number of missing values	0				
